# Historical Statement of the Galvanic Discovery, and of the Publications Which Have Appeared on That Subject

**Published:** 1803-07-01

**Authors:** 


### Historical Statement *of the* Galvanic Discovery, *and of the* Publications *which have appeared on that* Subject.

[ Continued from Vol. vii. p. 161. ]

*Observations on the Influence which produces Contraction in the Muscles of Animals, agreeably to Mr. Galvani's Experiments, by Dr. W. C. Wells; see Philosophical Transactions for the Year* 1795, *part ii. p.* 246, *translated in Gren's Journal der Physik, vol. iii. No.* 4.

The author endeavours to answer the following questions: 1. Does the incitement of the influence which in Mr. Galvani's experiments, occasions the muscles of animals to contract, either wholly or in part depend upon any peculiar property of living bodies? 2. What are the conditions necessary for the excitement of this influence? 3. Is it electrical? With respect to the first question, the author is inclined to answer it in the negative. Having employed various moist substances, in which there could be no possibility of the existence of life, to constitute with one or more metals, different from that of the coatings of the muscles and nerve, a connecting medium between those coatings, he found that they produced the same effect as his own body. He had accidently observed, that on covering a muscle and its nerve with two pieces of the same metal, the muscle was immediately thrown into contraction, if he applied the metal which he had used as a connector, and still held in his hand, to the coating of the muscle only, while with the other hand he touched the similar coating of the nerves; a curious circumstance, as it is known that no motion will take place, upon connecting those pieces by means of one or more different metals. A single drop of water was sufficient for the above purpose, though, in general, the greater the quantity of the moisture which was used, the more readily and powerfully were the contractions of the muscle excited. Concerning the second question, he remarks, that, without knowing the previous discovery of Mr. Volta, he found that charcoal is endowed with the property of exciting the Galvanic influence. The fact is, that as far as the contraction of the muscles is a test, whether the influence exists or not, and we have no other, it is never excited, when two metals, or one metal and charcoal are necessary for this purpose, unless the substances touch each other, and also are in contact with some of the fluids. But there is still another requisite for the excite ment of the influence, which is a communication, by means of some good conductor of electricity, between the two quantities of fluid, to which the dry exciters are applied, besides that which takes place between the same quantities of fluid, when the dry exciters are brought in contact with each other. Dr. W. likewise discovered that an exciting power may be given to a metal by rubbing it on many substances beside another metal, such as silk, woollen, leather, fish-skin, the palm of the human hand, sealing wax, marble, and wood. He thinks it probable, that the part which has been rubbed, is so far altered in some condition or property as to be affected differently by the fluid exciters from a part which has not been rubbed; in short, that the rubbed part becomes as it were, a different metal. The last question he answers in the affirmative, and thus expresses himself: "The points of difference between any two species of natural bodies, even those which from the similarity of some of their most obvious qualities, have once been thought the same, are found upon accurate examination, greatly to exceed in number those of their agreement. When, therefore, two substances are known to have many properties in common, while their differences are few, and none of these absolutely contradict such a conclusion, we infer with considerable confidence, that they are the same, though we may not be immediately able to explain why their resemblance is not complete.

*Extract of a Letter of Mr. J. Fabbroni in Florence.* In Crell's Chemischen Annalen, 1795, vol. ii. pp. 502.

The author is persuaded by the experiments which he made, that several phenomena, ascribed to this electricity, originate from a chemical power, which the dry and cold metals mutually exert, in order to decompose moisture or the *lympha animalis.*

*Vorlaufige Bekanntmachung der Natur des Metallreizes;* i. e. Preliminary Account of the Nature of the Metallic Stimulus, discovered by Prof. Creve of Mayance. In Hartenkeil's Medical and Surgical Gazette, (in German) 1796, vol. i. p. 49.

*Beytrage zu meiner Ankundigung, die Natur des Metallreizes Betreffend;* i. e. Additions to my previous Account, concerning the Nature of the Metallic Stimulus, by Prof. Creve, vol. i. p. 324.

Mr. Creve is of opinion, that on applying metals to animal parts, a chemical process takes place, by means of which the two metals decompose the water, and at the same time time disengage a portion of caloric, which combines itself with the caloric that is made free during the decomposition of the water. The oxygen unites itself afterwards with the metal and the hydrogen with the caloric, from which last combination arises an electrical matter, which being the product of this process, properly forms the proximate cause of the metallic stimulus.

*Ueber die gereizte Muskelfaser;* i. e. on the irritated Muscular Fibre; from a letter to Prof. Blumenbach, by Mr. Humboldt. In Gren's Neuem Journal der Physik, vol. ii. No. 2, p. 115.

From another letter of the same to the same; ibid. No. 4. p. 471.

*Versuche uber den Metallreiz, besonders in Hinsicht auf die Verschiedenartige Empfanglichkeit der Thierischen Organe;* i. e. Experiments on the Metallic Stimulus, particularly with respect to the different Sensibility of Animal Organs; from a letter to Prof. Blumenbach, by Mr. Humboldt, Ibid. vol. iii. No. 2.

*Versuche Uber die gereizte Muskel und Nerven Faser, nebst Vermuthungen uber den Chemischen Process des Lebens in der Thier und Pflanzen Welt;* i. e. Experiments on the irritated Muscular and Nervous Fibre, together with Conjectures on the Chemical Process of Life in the Animal and Vegetable World, by Frederic Alexander von Humboldt, tom. i. and ii. Posen and Berlin, for Decker and Rottman.

In stating the labours which this excellent Naturalist has bestowed on the Galvanic discovery, we need only give an account of the last work, in which his former notices are repeated, corrected, and enlarged. It comprises every thing relative to the Galvanic discovery, which was known to the time of its appearance, containing besides a rich store of new discoveries, interesting views, and observations, by which it is justly entitled to the attention and study of naturalists. The author has every where endeavoured to relate his observations and experiments, unprejudiced by the seducing train of hypothesis, and he modestly delineates the theory which he has formed on the nature of the Galvanic phenomena. We shall now proceed to a short analysis of the work itself.

First Part, Sect. I. The author prefers the expressions of *Galvanic stimulus,* to *Galvanise; Galvanismus,* as more proper than the denomination of *metallic stimulus;* and animal electricity, for the purpose of denoting the phenomena which Mr. Galvani has discovered in the nervous fibre, inserted in the muscle. The Galvanic agent only acts on the living nature, and is only perceptible in the matter which is endowed with the sensible fibre. It requires the re-action of a living animal fibre, and comprehends what is generally called vital action. No experiments have hitherto been able to make the action, which the Galvanic experiment most probably exerts on dead nature, combined with the excitable substances perceptible to our senses. The experiments which Mr. Humboldt undertook with this view, proved likewise without success. The success of the Galvanic experiments depends as well on the excitability and sensibility of the animal organs, as on the intensity of the stimulus, on which account it is of great importance for a proper examination of the Galvanic laws, to make the experiments on very sensible animals. With this view the author took frogs for his experiments, which were particularly successful. By bathing the nerves of these animals in alkaline solutions and in supersaturated muriatic acid, he was enabled to increase or diminish, at pleasure, the excitability of the fibre, and to resuscitate the languishing sensibility of animals in an artificial manner. As every stimulus debilitates the organs when too frequently applied, and as common acids applied to the nerves diminish the incitability, it was an easy thing for him to bring every animal substance from the highest degree of sensibility down to the lowest debility, direct or indirect. In order to examine the conditions and laws by which the muscular motions are produced in Mr. Galvani's experiments, Mr. H. distinguishes the state of sensibility, when naturally high or artificially increased, and when it is by any means diminished.

Sect. II. When the living irritable as well as sensible fibre is in a state of a naturally high or artificially increased sensibility, the muscular motion is observed to take place under two conditions; 1. if a fresh animal substance; or, 2. if metal or charcoal forms the conductor of the Galvanic fluid between the muscle and the nerve. In this section the first condition is considered; and the author excited muscular contractions by bending the thigh of a frog towards the ischiadic nerve, with which it was connected, while the crural nerve and muscle were touched at the same time by means of a piece of crural nerve, that had been cut out; by this method the animal's parts formed a connection, and conducted the influence from one part of the nerve to another.

Sect.

Sect. III. The second condition, by which, in the state of increased irritability contractions ensue, always requires the concomitant action of metalline or carbonised substances; but the author here distinguishes again two cases, viz. excitation occasioned by one metal or by homogeneous metalline parts, and excitation from heterogeneous metals. Homogeneous metals, causing muscular contractions, are applied to the animal organs in such a manner as to form a chain, or this chain does not take place. The author made several experiments relative to this different disposition of the exciters; and among others, we shall only point out the first experiment, grounded on the last case, where no chain is formed. Mr. H. having armed the crural nerve of a very lively frog with zinc, was proceeding to connect this coating and the crural muscle by means of another zinc wire; but the two metals had hardly been brought in contact with each other, without the zinc wire having touched the muscle or nerve, when most violent contractions ensued. It has been questioned whether homogeneous metals could excite contractions as muscular or as nervous coatings. In order to decide this question, Mr. Humboldt having poured pure quicksilver into a porcelaine saucer, and prepared the thigh of a frog in such a manner, that in a horizontal position of the thigh the crural nerve and a bunch of the muscle hung down, the leg was tied to a glass tube which was kept horizontally suspended over the saucer; and when the nerve touched the surface of the quicksilver, no contraction was perceived; but the muscle, on being brought in contact with the quicksilver, was convulsively contracted.

Sect. IV. Contractions of the muscles ensue if the nerve as well as the muscle are coated with heterogeneous metals, and either immediatly touch each other or through the medium of a moist conducting body; if homogeneous metals are applied to the muscle and nerve, and connected by a heterogeneous metal; if homogeneous metals coat the muscle and nerve, connected by means of two moist substances and one heterogeneous metal; if in the chain between the nerve and muscle several metals alternate with pieces of muscular flesh, and if there is only one homogeneous metal among the rest, &c. When the living organs are in a state of diminished incitability, they require a more violent stimulus, in order to produce muscular contractions.

The Galvanic phenomena, then, appear under two principal cipal conditions, that the heterogeneous coatings of the nerves and muscles touch each other immediately, or by means of exciting intermediate substances; and that homogeneous muscular and nervous stimuli are connected by a heterogeneous metal, the surface of which is to be covered with an evaporating fluid. This last property the author discovered by a singular experiment, which he here relates. He likewise mentions some experiments, according to which, animal parts appear to possess a conducting atmosphere. On placing, instead of the fluid, a piece of the muscle on zinc, lying on the gold plate, with which the nerve was armed, he not only perceived the convulsions on touching the muscular flesh, but on removing it to the distance of $\frac{1}{4}$ line from the gold plate.

Prof. Grimm, of Liegniz, in Silesia, who from the first discovery of the Galvanic agens, thought of employing it with a medical view, has published the results of his experiments in a medical journal, entitled, Archives of Practical Medicine for the Provinces of Silesia and South Prussia, (in German). He observed several disagreeable symptoms to arise from the application of Galvanism too long continued, viz. weariness, diarrhœas, and catarrhous complaints. He employed Galvanism in dimness of sight, and in difficulty of hearing; in the first complaint without avail, while in the latter it frequently proved successful. He relates one case of obstinate ophthalmia which he succeeded in curing by bringing the patient in connexion with the Galvanic chain, for which purpose he placed the zinc wire on the tongue, letting the patient at the same time touch the uppermost copper-plate with a staff of iron, the hand in which he held it being previously moistened. This case proves the great influence of the Galvanic agens on the organs of sight, though the neighbouring parts are only acted upon. Prof. G. recommends the Galvanic apparatus of Mr. Cruikshank, which has the advantage of keeping its efficacy for several weeks without requiring to be cleaned and rubbed, which is always a very tedious operation.

Dr. Zadig, of Breslau, was so fortunate as perfectly to cure a man of sixty years of age, who was affected with amblyopia by the use of Galvanism continued for about six weeks. He began with ten strata of plates, gradually increasing that number to thirty. He placed the copper wire into a glass bowl with salt water, in which the patient was ordered to immerse his hands, while the doctor touched ed the upper eye-lid with the zinc wire, which he repeated twice a day for fifteen or twenty minutes.

In the *Annalen der Physik*; i. e. Annals for Natural Philosophy, published by Prof. Gilbert of Halle, Mr. Emhoff of Celle communicates his experiments relative to the medical application of Galvanism on persons affected with difficulty of hearing, which in a great measure seems to arise from a rheumatic cause, as the complaint increases in bad and rainy weather; but these experiments did not answer his expectation. In one case, the application of Galvanism was continued every day for six weeks, without producing any favourable effect, though a weak degree was first employed, and the Galvanic shocks gradually increased, so as to make them very sensible to the patient.—Another patient thought himself relieved for a short time; but though he was galvanised with a pile of 100 strata, he perceived not the least effect from it. The experiments however, which Mr. E. made on four persons who were deaf and dumb proved more satisfactory. In these cases he made use of a pile, consisting of 100 strata of zinc and copper plates with round pieces of felt which he had dipped in very diluted nitric acid; taking twelve parts of water to one part of the acid, which he found of all wet conductors to be the most advantageous. At the two ends of the pile brass wires were adapted, ending in small hooks, to which the brass conductors were fastened, by means of which the Galvanic shocks are carried to the ear. For the purpose of insulating them, they pass through glass tubes; some of them are pointed, while others are provided with a small knob either of lead or of wood, by which means Mr. E. was enabled to increase or diminish the effect of the pile, according to the different indication, because the pointed conductors are the most efficacious, and those which have a wooden knob show the weakest action. Before the conductors are applied, Mr. E. moistens the concha and external parts of the ear with water of ammonia, and introducing one wire into the ear, he touches the other ear with the opposite conductor twice or three times within a second minute. The patient could be exposed to a greater or less degree of Galvanism, as he increased or diminished the number of the strata. The patients on which Mr. E. used the Galvanism in this manner, were, a boy twelve years old, who had lost his hearing in the small-pox in the fourth year of his age; a girl aged eight, born deaf; and two adults; in all which cases the application tion proved successful. The boy was within two months so far advanced, that he could hear words spoken near him with a low voice; however, when his attention was engaged he seemed not to regard the loudest voice. He was particularly sensible to sounds which he was not accustomed to hear. He learned in a short time to pronounce the vowels, and by degrees the consonants, and some easy words. The effect of Galvanism was still more striking with the girl, as she acquired after five weeks the full power of her organs of hearing; she heard the lowest voice, turning towards the person who speaks, and she begins to speak and babble as children do. In the adults on whom Mr. E. employed the Galvanism, the cure advanced more slowly; however, after an application continued for six weeks, they were able to distinguish the sound of a bell, of drums, of a flute, &c. which induced Mr. E. to continue the practice, the results of which he has promised to communicate to the public.

Dr. Martens, of Leipzig, has likewise made a series of experiments on the medical application of Galvanism, which he has published in a medical journal, entitled Parodoxien, of which he is the editor. In four cases of suppressed gonorrhœa venerea, where the scrotum began considerably to swell, he re-established the running by means of Galvanism, and thus removed the swelling. For this purpose he introduced a silver probe into the urethra, bringing it in connection with the zinc pole of a Galvanic battery, which consisted of 12 strata. To the copper pole he fastened a silver spoon, which he applied to the perinæum, which had been previously moistened with a solution of sal ammoniac. The experiment was repeated only twice within five minutes, and occasioned a painful sensation in the affected part.

In three cases of paralytic diseases, Dr. M. employed the Galvanism with much success.

1. In a paralysis of the upper eye-lid, which he removed in three days, by means of a battery of 12 strata. On the two first experiments no sensible effect could be perceived, but during the third experiment the paralysis suddenly disappeared.

2. In a paralysis of the extensores digitorum, which was removed by the application of Galvanism fifteen times repeated. The battery consisted in this case of 35 strata. He fastened a zinc plate on the metacarpus of the thumb, and a copper plate on the fifth metacarpus, placing under each each a piece of cloth wetted with a solution of sal ammon. and he brought them alternately in connection with the battery.

3. In an imperfect paralysis of the thigh, which had lasted for above year. He put a blister near the trochanter, and another below the flexion on the anterior part of the thigh, keeping the sores open by means of Galvanism. The patient found himself greatly relieved, but the cure was not completed, when the author published his account.

In a dimness of sight the Galvanism proved likewise efficacious, but without removing the affection entirely.

The faculty of hearing and speaking in a person born deaf and dumb, who could only distinguish some kinds of sound, was likewise much improved by the use of Galvanism; the results however were uncertain, as the patient was prevented from continuing it for a proper time.

In several cases of difficulty of hearing, Galvanism was also employed with success. Dr. M. relates eight such cases, in all which the patients experienced great relief. For moistening the cloth he thinks a solution of sal ammon. to be the most efficacious, but salt water preserves its efficacy much longer. The best composition for that purpose consists in the following mixture, ℞. Sal commun. unc. j. fell. taur. recent. unc. j. aq. font. unc. iv. tinctur. heliotrop. dr. j.

Dr. Marten's method of applying Galvanism in affections of the auditory organs is the following:

1. If deafness be equally strong in both ears, he conducts the zinc wire into the one, and the copper wire into the other ear, but he changes the application, alternately introducing the zinc wire and copper wire into the ears.

2. If one ear be more affected with deafness, he always conducts the zinc wire into that ear.

3. Should deafness merely reside in one ear, he applies the zinc wire to the affected ear and the copper wire to the sound, moistening the linen in which the zinc wire is wrapped with a solution of sal ammon. whereas the copper wire is moistened only with common water. In this case the patients have scarcely any sensation in the sound ear, while the Galvanic agens is fully perceptible in the affected ear. Or he orders the patient to immerse the hand of the side opposite to the affected ear in a bowl with salt water, into which the wire of the copper pole has been conducted; a method far preferable to the former.

Dr.

Dr. Martens disapproves of the different machines proposed for fastening the conductors in the ear by Drs. Augustin, Grapengiesser, and Bishoff, as being too compound, expensive, and inconvenient. He prefers a machine invented for that purpose by Dr. Tittman of Leipzig, which consists of four different parts, namely a bandage to be tied round the head, a hoop of whalebone, two small wood plates to be applied above the ears, and two small pieces of wood, one inch long and half an inch in breadth. The bandage is about two inches in breadth, and is made of double cloth, which can be buckled round the head. On both sides, above the ears, are fastened two small boards three inches long, one and a half broad, and half an inch thick, longitudinally perforated with thirty holes at the same distance from each other. At the upper margin, two larger holes are made for fastening the hoop that is bent over the head. The above mentioned pieces of wood have each a large hole, through which the conductors pass, and at their upper margin strong iron wire is fastened, pointed and bent in a right angle. This machine is tied round the head above the eyes. The conductors are passed through the holes in the small pieces of wood, which are fastened by means of the iron wire into the holes of the boards, and are to be placed higher up or lower down, as may be found convenient. Another apparatus, still more simple, is proposed by Dr. Martens, of which he gives the following description: Take two round pieces of cork, one inch in diameter and half an inch thick; push through the centre of each a thin silver wire, three inches long; make on each side, at equal distance from the centre, a hole through the narrow part of the round pieces of cork, through which draw silk strings, each two yards long. Thus the two cork pieces may be placed on the ears, by tying the string under the chin. One part of the silver wire, which reaches into the ear, is wrapped in a little linen, while the other, which is on the outside, is bent as a ring or hook, on which the conducting chains are fastened. The linen, sewed round one end of the wire, must be moistened with salt water before it be introduced into the ear. This apparatus is both simple and convenient, and suits to every head.

[ To be continued. ]

EXPERIMENTS

